# The effects of individual music therapy in nursing home residents with dementia to improve general well-being: study protocol of a randomized controlled trial

**DOI:** 10.1186/s12877-024-04863-z

**Published:** 2024-03-27

**Authors:** Vanusa M. Baroni Caramel, Jenny T. van der Steen, Annemieke C. Vink, Sarah I. M. Janus, Jos W. R. Twisk, Erik J. A. Scherder, Sytse U. Zuidema

**Affiliations:** 1 Department of primary and long-term care, University of Groningen, University Medical Center Groningen, Groningen, The Netherlands; 2 Faculty of Behavioral and Movement Sciences, Clinical Neuropsychology, Amsterdam, The Netherlands; 3 Department of Public Health and Primary Care, Leiden University Medical Center, Leiden, The Netherlands; 4 Department of Primary and Community Care, Radboud university medical center, Nijmegen, The Netherlands; 5 Department of Music Therapy, ArtEZ University of The Arts, Academy of Music, Enschede, The Netherlands; 6 Department of epidemiology & data science of Amsterdam UMC, Amsterdam, The Netherlands

**Keywords:** Dementia, Quality of life, Psychosocial intervention, Music therapy, Behavioral symptoms, Randomized controlled trial, Nursing homes, Neuropsychiatric symptoms

## Abstract

**Background:**

Dementia is often associated with Neuropsychiatric Symptoms (NPS) such as agitation, depression, hallucinations, anxiety, that can cause distress for the resident with dementia in long-term care settings and can impose emotional burden on the environment. NPS are often treated with psychotropic drugs, which, however, frequently cause side effects. Alternatively, non-pharmacological interventions can improve well-being and maintain an optimal quality of life (QoL) of those living with dementia. Other QoL related outcomes, such as pain, discomfort and sleep disruption are relevant outcomes in music trials as well. Music therapy is a non-pharmacological intervention that can reduce NPS and improve well-being, and its associated symptoms in dementia.

**Methods:**

The research will be conducted at eight nursing home facilities of a health care organization in the Netherlands. A sample size of 30 in each group (experimental and control group) is required, totalling 60 residents increased to 80 when considering expected drop out to follow up. The participants in the intervention group receive 30 min of individual music therapy (MT) in their own room by a music therapist twice a week for 12 weeks. The participants in the control group will receive 30 min of individual attention in their own room by a volunteer twice a week for 12 weeks. Assessments will be done at baseline, 6 weeks and 12 weeks. An independent observer, blinded for the intervention or control condition, will assess directly observed well-being (primary outcome) and pain (secondary outcome) before and after the sessions. Nurses will assess other secondary outcomes unblinded, i.e., perceived quality of life and NPS, both assessed with validated scales. The sleep duration will be indirectly assessed by a wrist device called MotionWatch. Information about psychotropic drug use will be derived from electronic medical chart review.

**Discussion:**

The main purpose of this study is to assess the effects of individual music therapy on directly observed well-being controlled for individual attention in nursing home residents with dementia with NPS. The outcomes refer to both short-term and long-term effects consistent with therapeutic goals of care for a longer term. We hope to overcome limitations of previous study designs such as not blinded designs and music facilitators that were not only music therapists but also occupational therapists and nurses. This study should lead to more focused recommendations for practice and further research into non-pharmacological interventions in dementia such as music therapy.

**Trial registration:**

The trial is registered at the International Clinical Trials Registry Platform (ICTRP) search portal in the Netherlands Trial Registration number NL7708, registration date 04-05-2019.

## Introduction

### Background and rationale

Dementia is a common disease and in 2021, the World Health Organization estimated that around 55 million people have dementia worldwide [1]. Dementia is a neurodegenerative syndrome characterized by a progressive deterioration of cognitive function, in particular memory but other cognitive domains such as language, praxis, visual perception and most notably executive functions are also often affected [2]. Personality and behavior changes, together with a decline in the ability to perform activities of daily living, may result in a loss of independence. The personality and behavioral changes are often reflected in Neuropsychiatric Symptoms (NPS). More specifically, NPS may express itself in, for example, agitation, disinhibition, irritability, delusions, hallucinations, depression, anxiety and apathy. These symptoms are ubiquitous in nursing home patients with dementia, with overall rates of more than 80% [3].

As there are no curative treatments for dementia yet, it is important to focus on interventions that may have beneficial effects on well-being and maintain an optimal quality of life. Quality of life is defined by the WHO as “individuals’ perceptions of their position in life in the context of the culture and value systems in which they live and in relation to their goals, expectations, standards and concerns” [4]. Well-being is defined in terms of a state of equilibrium existing between personal resources and life challenges that, when achieved, gives rise to positive emotions and psychological health [5]. The constructs of QoL and well-being have often been used inter-changeably in dementia research [6].

There is a growing consensus that quality of life is an important outcome for assessing the effectiveness of interventions for dementia in clinical trials even though it concerns a multidimensional construct influenced by a variety of factors [7, 8]. An observational study in 288 nursing home residents with dementia, showed that NPS such as agitation and depression were particularly strong predictors of poor QoL [9]. These results underline the growing awareness that NPS independently decreases QoL in moderate and severe stages of dementia [9]. Pain is highly prevalent in nursing home residents [10, 11] and can also cause NPS and influences their quality of life directly [12]. Nursing home residents with severe cognitive impairment who have difficulty expressing pain may manifest it through agitation, aggression, or withdrawal [10]. Pain in dementia may express itself also through vocalizations (e.g., crying, screaming, noisy breathing), specific facial expressions (e.g. grimacing) and body language such as restless behaviour [13, 14].

Another factor that may reduce QoL of people with dementia is a deterioration of sleep quality. Sleep disturbances include poor sleep efficiency and increased night awakenings. Petrovsky [15] used domains of Lawton’s framework for quality of life in persons with dementia to synthesize current knowledge on the association between sleep disruption and quality of life in persons with dementia. Sleep disruption was negatively associated with all of four QoL domains (physical functioning, social/behavioral functioning, emotional well-being and cognitive function). In sum, neuropsychiatric symptoms, in relation to or provoked by pain and sleep disturbances, reduces QoL in people with dementia.

Consequently, the question arises if adequate strategies are available for treating this triad of symptoms, i.e. NPS, pain, and sleep disturbances, and hence, improving QoL, in people with dementia. NPS are often treated pharmacologically with psychotropic drugs, which frequently cause unwanted side effects, such as somnolence and extrapyramidal symptoms [16]. Psychosocial interventions, however, may obviate the indication for antipsychotic drug prescriptions [17]. Moreover, therapies such as validation, reminiscence, psychomotor therapy, multisensory stimulation and music therapy can increase QoL of people with dementia and their caregivers [18]. Sikkes et al. reviewed the evidence and found that various non-pharmacologic treatments such as music therapy effectively improve behavior [19].

Music therapy is a non-pharmacological intervention which is used as a treatment for NPS in patients with dementia [20–22]. In the USA, music therapy is defined by the American Music Therapy Association (AMTA) as “the clinical and evidence-based use of music interventions within a therapeutic relationship to accomplish individualized goals by a credentialed professional who has completed an approved music therapy program” [23]. This definition of music therapy and the accompanying AMTA Standards of Practice recommend an individualized treatment process, including referral, building a therapeutic relationship, assessment, observation, targeting individualized goals and objectives, treatment planning, protocol selection and implementation, termination and evaluation [24]. The number of studies examining the effects of music therapy for people with dementia has increased over time [25]. A Cochrane review in 2011 [26] included 10 studies mostly of poor quality and could not draw firm conclusions about the effects of music therapy in the treatment of behavioral, social, cognitive and emotional problems of older people with dementia. In an update of the Cochrane review [27] twenty-two studies were included. The results from the review suggest that music therapy may also improve emotional well-being including quality of life. However, outcomes could be based on recall or direct observation, and not all outcomes are assessed blinded. The results of the review further suggest that providing institutionalized people with dementia with at least five sessions of a music-based therapeutic intervention might reduce NPS. Moreover, individual therapy, compared with group therapy, had larger effects on behavioral outcomes (agitation, aggression and overall behavioral problems).

Indeed, music therapy can be offered via individual treatment, as well as through a group approach [28]. The group approach benefits engagement and social interaction but in advanced stages of dementia, individual music therapy can better reach the residents with communicative limitations. Personalized interventions with music or music therapy may be a predictor of success [20].

In 2010, we conducted a randomized pilot study on individual music therapy in a nursing home in the Netherlands. The intervention group received individual music therapy from a qualified music therapist who used a person-centered approach. The results pointed to possible reduction of NPS in patients who received individual music therapy compared to patients in the control condition who received usual care [29]. The sample size of the pilot study was very small and the outcome assessments were not blinded. Sakamoto [30] conducted an RCT in 2013 with blinded outcome assessment and individual intervention sessions carried out by music facilitators. The study indicated that interactive individualized interventions reduce stress and increase relaxation in individuals with severe dementia immediately after the intervention.

### Objective

The main purpose of the present study is to build on the earlier pilot study with an improved design, in which we blindly assess the effects of individual music therapy delivered by an accredited music therapist on directly observed well-being and related outcomes such as NPS, agitation, depression, quality of life and pain controlled for individual attention in nursing home residents with dementia who also have NPS.

### Trial design

The design involves an individual randomized controlled trial (RCT) employing longitudinal repeated measurements in nursing home residents with dementia and NPS. The study is single blinded. The research assistant who assesses the primary outcome well-being and the secondary outcome pain through observation does not know whether residents participate in the experimental group or the control group. The music therapist and the patients themselves cannot be blinded to the condition they are assigned to. Nursing staff that performs the measurements as part of the secondary outcomes is not blinded either. The research takes place at eight nursing homes facilities of a health care organization in the Netherlands. Music therapy is provided in 30-minutes sessions, twice a week for 12 weeks, in their own room. The control group receives a ‘social’ visit with individual attention with the same frequency and of the same duration.

A process evaluation is performed according to an approach developed by Saunders [31], using components from Linnan and Steckler’s [32]. The process evaluation is based on mixed methods, collecting quantitative and qualitative data. Qualitative data collection comprises a focus group discussion with participants of health care professionals to evaluate barriers and facilitators influencing the implementation of research protocol. Quantitative data is gathered with questionnaires about reach, dose delivered, dose received, fidelity, recruitment and participant engagement. Quantitative data is gathered with questionnaires at 1, 2, 6 and 12 weeks after the baseline assessment completed by music therapists (intervention) or attendants (control). The questionnaires include items about participation in the sessions, fidelity, dose, engagement and about levels of implementation. Nurses and a research assistant will fill a questionnaire at the end of treatment to evaluate the process of implementation. Quantitative data will be analyzed with descriptive statistics.

## Methods

### Study setting

The study population consist of a sample of residents with dementia and NPS, residing on psychogeriatric units in one of eight nursing homes facilities of a health care organization (Amstelring) in the Netherlands.

### Participants’ eligibility

#### Inclusion criteria

First, potentially eligible participants of psychogeriatric units in eight nursing homes are screened for eligibility according to the following criteria:

1. Candidate has a charted diagnosis of dementia, which is in general according to Diagnostic and Statistical Manual of Mental Disorders IV criteria (American psychiatric association, 2001).
2. Display a clinically relevant NPS measured with the Neuropsychiatric Inventory Nursing Home Version (NPI-NH) [33, 34] with the Frequency X Severity item score for at least one individual item rated 4 (Wood, 2000 [35]; Margallo, 2001 [36], Zuidema, 2007 [3], Zuidema, 2010 [37].

#### Exclusion criteria

1. Candidate having received individual music therapy before or having participated in a music therapy group in the past 3 months.
2. Candidate has major comorbid psychiatric diagnosis (i.e., schizophrenia, psychosis, anxiety disorder). Due to an overlap between depression and dementia [38], candidate participants with a history of depression will not be excluded.
3. Candidate has a hearing impairment that hampers the listening to music at a moderately volume. We use item 1a of the Severe Dual Sensory Loss in old age screening tool (SDSL) [39]. SDSL was found a valid and reliable tool [40].

### Demographic variables

Demographic variables such as age and gender will be assessed at baseline from the electronical medical chart review.

At baseline, nurses will register whether the resident has any background in music with questions about their experiences with music before entering the nursing home. The relatives of participants are asked closed-ended questions if the participant:

1. Has no experience with music in the past.
2. Played an instrument before or was involved in singing activities or made music in some other way.
3. Has served as a professional musician.

## Material and procedure

### Intervention

The participants of the intervention group (music therapy) receive 30 min of individual music therapy twice a week for 12 weeks in their own room. Music therapists use music experiences to promote health [41]. In this trial, music therapy consists of individual active music therapy sessions with also receptive techniques. Individual music therapy implies a one-to-one contact between the music therapist and the patient using a person-centered approach. Receptive and active music therapy are often combined [42]. Receptive therapeutic interventions consist of listening to music by the therapist who sings, plays, or selects recorded music for the recipients. In active music therapy, recipients are actively involved in the music-making, for instance by playing on small instruments. The participants may be encouraged to participate in musical improvisation with instruments or voices, movements activities or singing.

To determine the precise content of the intervention and to standardize procedures amongst all music therapists of Amstelring we conducted a focus group with 7 music therapists working at the nursing home facilities of Amstelring. We used a group interview because reaching a consensus is best achieved in a group setting. The focus group established a therapy protocol that comprised six steps for this study purpose:

1. All participating music therapists are employed by the nursing home organization. The music therapists are qualified and credentialed professionals by the federations for music therapy in the Netherlands (NVvMT), indicating that they have all been trained at an acknowledged and certified music therapy study program.
2. The music therapist will ask, before the start, the participant or a legal representative about the musical preferences of the participant with a questionnaire (Gerdner, 2010 [43], Raven-de Vries, 2018 [44]). Based on this information, the music therapist will develop an individual music therapy session for the participants.
3. Music therapists start each session trying to connect with the patient by eye contact, calling the name or giving a hand, to build trust.
4. Music therapists use an observation list between 4 and 6 weeks. This observation list is based on the improvisational music therapy guideline of Kurstjens [45] and has been used to identify which important musical elements are of help in the approach of the problem.
5. The music therapists will adapt the therapy continuously to needs and wishes of the participants. Participants receive an individual session tailored to the musical preferences of the participant.
6. The participant will be invited to make a choice out of different musical instruments such as a guitar, keyboard, drums, maracas, etc. The therapist will aim at using active techniques mainly, for example improvisation where participants will try to play the instruments, sing together, clap the hands, move the body or face with the music. Also, receptive techniques will be used such as listening to live or pre-recorded music. The music therapist provides a safe environment, in which the participant can experience contact, interaction, atonement, structure and a natural finishing of the session.

In case of refusal of the session of adverse reactions such as fatigue or traumatic memory during the sessions, music therapists inform the researcher (VMBC). The music therapists then stop immediately. Music therapists fill in questionnaires at 1, 2, 6 and 12 weeks after the intervention starts to gather information for the process evaluation. During the Covid-19 outbreak, the music therapists (intervention group) use gloves and face masks during the sessions.

### Control condition

The control group receives 30 min of individual attention twice a week for 12 weeks with no therapeutic basis. Individual attention will take place through a social visit, during which an attendant will drink coffee or tea with the participant. The individual attention will be given in the resident’s room by one attendant, who will have a conversation with the participant, drink coffee without an intervention goal such as cognitive training and without any musical intervention. The person who is doing the social visits is an informal care support company employee or volunteer. The attendants are students who work for an informal care support company and had a workshop about dementia. This is on top of the regular usual care delivered. The attendant starts every session by trying to connect with the patient via eye contact, calling his/her name or giving a hand, to build trust.

The attendants from the informal care support company or care support volunteer who provide the individual attention report attended sessions in the participants forms. In case of refusal of the session, the attendants inform the researcher (VMBC). The individual attention will then stop immediately. Attendants fill in questionnaires at 1, 2, 6 and 12 weeks after the individual attention starts to gather information for the process evaluation. During the Covid-19 outbreak, the attendants (control group) use gloves and face masks through the sessions.

### Primary outcome measure

Observed well-being is the primary outcome measure rather than multidimensional quality of life with proxy estimation of domain ratings based on recall. Lawton has most extensively explored the concept of QoL concerning dementia and described QoL in dementia in terms of four sectors: psychological well-being, behavioral competence, objective environment (response to surroundings) and perceived QoL [46–48]. Jonker et al. reviewed conceptual developments in QoL research concerning dementia and based on the dimensions presented by Lawton, they identified psychological well-being as the core dimension of QoL of patients with dementia. Patients with severe dementia cannot always self-report their psychological well-being [49] but indicators of wellbeing of persons with dementia can be observed with validated instruments. We assess well-being with an objective observational instrument−the Discomfort Scale - Dementia of Alzheimer Type (DS-DAT) where discomfort is defined as a negative emotional and/or physical state subject to variation in magnitude in response to internal or environmental conditions. It is a scale for direct observation of behavior of a patient and is applicable also in later phases of dementia [50]. The Dutch translation is characterized by a good reliability (Hoogendoorn. 2001) [51] and validity (van der Steen, 2002) [52, 53]. The scale consists of 9 items measuring 7 negative and 2 positive items regarding vocalization, breathing, facial expression, and body movements. The nine 4-point items are summed for a total score ranging from 0 (no observed discomfort) to 27 (highest possible level of observed discomfort). Well-being will be assessed by a blinded research assistant at baseline (T0), six weeks (T1) and twelve weeks (T2). Well-being is assessed during 5 min before and after the music therapy sessions or individual attention sessions. The research assistant will be trained in using the DS-DAT trough an instructional video and practice completion of the DS-DAT with videotaped patients. Feedback is provided against the scores of the scale’s developer (Dr Hurley) who had rated the patient video clips.

### Secondary outcomes measures

Secondary outcomes are also assessed in all /participating residents and include pain, quality of life, neuropsychiatric symptoms (agitation, anxiety, symptoms of depression), quality of sleep and psychotropic drug use.

**Pain** will be assessed by a blinded research assistant at baseline, six weeks and twelve weeks during 2 min before and after the intervention session or individual attention with the PAIC-15 (Pain Assessment in Impaired Cognition) [54, 55] which is an observational assessment instrument that lists 15 items and uses scores from 0 to 3 for each item. PAIC-15 comprises three domains: facial expression, body movements and verbalizations/vocalizations. Each domain has 5 items and the total score is the sum of all items, ranging from 0 to 45. The research assistant will be trained in using the PAIC-15.

**Quality of life** will be rated by non-blinded nurses at baseline (T0), at six weeks (T1) and at twelve weeks (T2) with the Quality of Life in Late-Stage Dementia Scale (QUALID) [56] that can be used with late-stage dementia patients in institutional settings and has been designed for proxy-rating by nurses. Responses reflect patient behavior over the past seven days. The QUALID consists of eleven items that are short and simple and is characterized by a good reliability and validity and the Dutch translation was a valid measure for quality of life in patients with advanced dementia [57]. The total score is the sum of all items, ranging from 11 to 55. Lower scores indicate a higher quality of life.

**Neuropsychiatric symptoms (NPS)** will be assessed by non-blinded nurses at baseline (T0), at six weeks (T1) and at twelve weeks (T2) with the Neuropsychiatric Inventory Nursing Home Version (NPI-NH), a scale originally developed by Cummings [33, 34] to assess NPS in outpatients with dementia. The nursing home version was developed for use of professional caregivers in institutions and proved to be valid and reliable for trained nursing staff [58, 59]. The NPI-NH is the only nursing home instrument to assess NPS that occurred in the past four weeks that has been translated into Dutch [60]. The NPI is a structured interview that includes 12 neuropsychiatric symptoms: delusions, hallucinations, agitation, depression, anxiety, euphoria, apathy, disinhibition, irritability, aberrant motor behavior, night-time disturbances and appetite/eating change. Frequency (F) and severity (S) of each symptom are rated on a four (1–4) and three (1–3) point scale respectively. A separate score can be calculated for each symptom by multiplying the frequency and severity (FxS score), resulting values range from zero to 12 for each symptom. Summing all FxS scores results in a total score that ranges from 0 to 144. Interview also includes a caregiver distress questions using a 5-point scale.

**Agitation and aggression** will be assessed by non-blinded nurses at baseline (T0), 6 weeks (T1) and 12 weeks (T2) using the caregiver rated questionnaire Cohen-Mansfield Agitation Inventory (CMAI). This instrument, developed by Cohen-Mansfield and Billig [61] and validated by Miller [62] is an instrument that specifically addresses agitation or aggression that has been translated into Dutch. The Dutch translation of the CMAI (CMAI-D) has been validated by De Jonghe [63] and rated agitated behaviors occurred during last two weeks. The frequency of each symptom is rated on a seven-point scale (1–7) ranging from ‘never’ to ‘several times an hour’. Summing all symptom scores results in a total score that ranges from 29 to 203.

**Depression** will be rated by non-blinded nurses at baseline (T0), at six weeks (T1) and twelve weeks (T2) with the Cornell Scale for Depression in Dementia (CSDD) [64], which has good internal consistency [65]. Responses reflect symptoms of depression in the week before. The CSDD is a 19-item instrument, with scores for each item ranging from 0 to 2 (total score range 0–38). The CSDD has been translated into Dutch [66].

**Physical activity**: rest-activity data will be collected by a MotionWatch (CamNtech Ltd, Cambridge, UK). The MotionWatch measures the arm movements of the participant; based on these movements, the rest-activity and physical activity are determined. It quantifies accelerations due to motor activity of the arm and integrates these over I-minute periods. The MotionWatch has the size and shape of a watch, is worn on the dominant wrist. The participants will be asked to wear the MotionWatch twenty-four hours a day for 1 week in the week before the intervention starts (T0) and the week directly after the last intervention session (T2). Nurses are asked to temporarily take off the MotionWatch when the participant takes a shower, performs another activity in which the MotionWatch could be exposed to too much water. Nurses will report the time that devices are taken off. Five parameters are calculated: Interdaily Stability (IS), Intradaily Variability (IV), Relative Amplitude (RA), most active period of 10 h and least active period of five hours. First, the IS variable that quantifies the strength of coupling between the rest-activity rhythm and supposedly stable Zeitgebers (e.g., meals) is calculated. IV, which quantifies the fragmentation of the rhythm, that is, the frequency and extent of transitions between rest and activity. RA quantifies the difference between the main activity (day) and rest (night) periods. The parameter of 10 most active hours (M10) is used to determine the amount of physical activity. The parameter (L5) are least active periods of five hours to determine rest activity.

Data on chronic psychotropic drug use will be derived from the electronic medical chart. Psychotropic drugs will be categorized into antipsychotics, anxiolytics, hypnotics, antidepressants and anti-dementia drugs according to the Anatomical Therapeutic Classification (ATC) system.

Any attrition or adverse effects of the interventions will be documented. The interventions will be discontinued if, in consultation with the physician or psychologists, harmful effects are observed and are expected to continue with exposure to any of the two interventions.

The nurses will determine the stage of dementia, measured with the Global Deterioration Scale (GDS) [67, 68] at baseline. The DS describes seven stages: ‘no global impairment’ (1), ‘very mild cognitive decline’ (2), ‘mild cognitive decline’ (3), ‘moderate cognitive decline’ (4), ‘moderately severe’ (5), ‘severe’ (6) and ‘very severe global impairment’ (7).

### Participants timeline

**Fig. 1:**
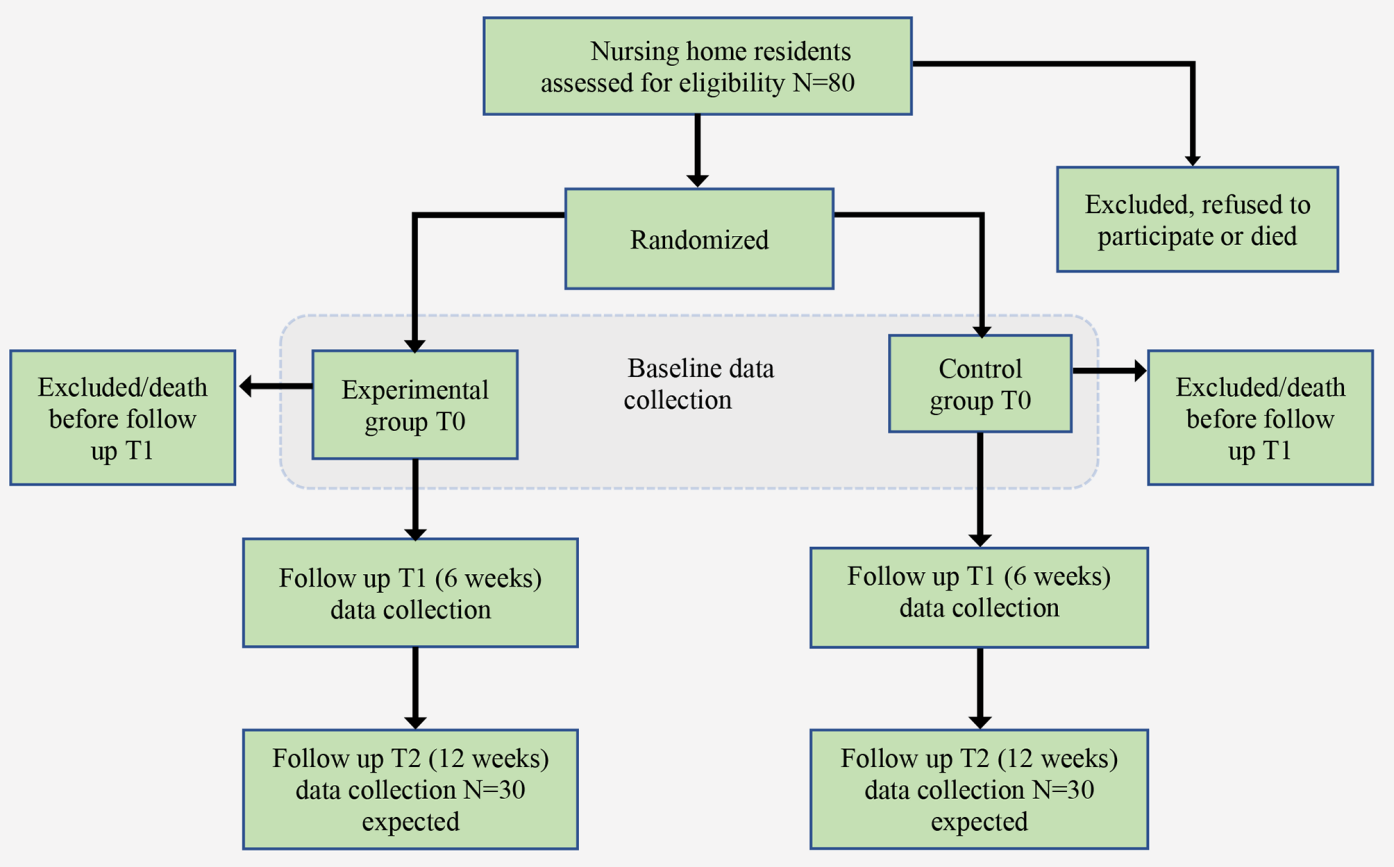
Participant flow: T0 baseline data collection, T1 follow up assessment at 6 weeks and T2 the follow up after 12 weeks. Participants who are lost to follow-up at the T2 assessment can still be included in the analyses, due to the use of mixed models analyses

All eligible residents for whom consent have been provided will be randomly allocated to the intervention group (individual music therapy) or control group (individual attention). Randomization will take place with a statistician using randomization software. The outcomes variables are measured at baseline (T0) one week before individual attention of music therapy starts (pre-treatment), after 6 weeks of intervention (T1) and after 12 weeks of intervention (T2). Figure 1 shows the participant’s flow.

Measurements (DS-DAT and PAIC-15) will be done at baseline T0, just shortly before (T1a and T2a) and shortly after the intervention (T1b and T2b); to assess overall effects, cumulative and immediate effects (**see** Table 1). An independent research assistant performs the blinded direct observations and complies the primary outcome well-being (measured with the DS-DAT) and secondary outcome pain (measured with the PAIC-15). The nursing staff will complete the unblinding observations scales concerning the various stages of dementia at baseline T0 and they will fill in the unblinding questionnaires about QoL, NPS, agitation and symptoms of depression at T1b and T2b.

Data prescription records concerning medication use, among which psychotropic drugs, will be extracted from the electronic medical charts by VMBC, physician employed by the participating organization.

**Table 1:** Instruments at different assessment moments

Outcomes/instruments	T0 week 0	T1a (pre-treatment) Week 6	T1b (post-treatment) Week 6	T2a (pre-treatment) Week 12	T2b (post-treatment) Week 12
Primary outcome					
Well-being (DS-DAT)	X	X	X	X	X
Secondary outcomes					
Pain (PAIC-15)	X	X	X	X	X
Quality of life (QUALID)	X		X		X
Neuropsychiatric symptoms (NPI-NH)	X		X		X
Agitation (CMAI)	X		X		X
Depressive symptoms (CSDD)	X		X		X
Stages of dementia (GDS)	X				
Rest/activity (Actigraphy)	X				X
Medication use (ATC classification)	X				X

DS-DAT: Discomfort Scale - Dementia of Alzheimer Type, PAIC-15: Pain Assessment in Impaired Cognition, QUALID: Quality of Life in Late-Stage Dementia Scale, NPI-NH: Neuropsychiatric Inventory Nursing Home Version, CMAI: Cohen-Mansfield Agitation Inventory, CSDD: Cornell Scale for Depression in Dementia, GDS: Global Deterioration scale, ATC: Anatomical Therapeutic Classification system

### Sample size

The sample size calculation is based on the study’s primary outcome measure, the Discomfort Scale – Dementia of Alzheimer type (DS-DAT) [51] for F-tests ANOVA repeated measures. To determine the sample size required to assess relevant effects, we used data from the Cochrane review on music-based therapeutic interventions [27]; the effect size for emotional well-being and quality of life (SMD 0.32, 95%CI 0.02 to 0.62). The correlation between multiple assessments of the DS-DAT was calculated with the data of Schalkwijk et al. [57] as 0.6. Using a type I error of 0.05, a power of 0.80, two follow-up measurements and 2 groups, a sample size of 30 in each group is required, totaling 60 participants to achieve 0.81 power. According to the availability of music therapists we estimated that the study would take approximately 3 years. The mortality rate in the nursing home setting is high. Moore [58] stated that the length of stay in long-term care facilities in Europe countries is almost 2 years. It is estimated that about a one third (20 of 60 residents) may die or will be lost to follow up for other reasons during the study period, so we need to enroll a total of around 70 till 80 residents for this study.

### Recruitment

First, the medical staff is informed about the goal and procedure of the study. Second, the medical staff (physicians) of each psychogeriatric unit of the eight nursing homes selects eligible residents with dementia and NPS who have a referral for individual music therapy. Third the researcher provides an information letter with informed consent form to competent residents and to legal representatives of non-competent residents. After informed consent or proxy informed consent has been given and the participant has been recruited, the participants are randomly allocated to the intervention group (individual music therapy) or control group (individual attention).

### Assignment of interventions

#### Allocation

Participants will be randomly assigned to either the control or the experimental group with a 1:1 allocation as per a computer-generated randomisation schedule. Randomization is performed by a statistician who is not involved in carrying out the study in the facilities, using randomization software.

#### Blinding

The study is single blinded, i.e. that the therapist and the patients themselves are not blinded. Trained research assistants who are blinded to intervention assignment, conduct the well-being (primary outcome) and pain (secondary outcome) measurements. The nursing staff completes the scales concerning the stage of dementia, QoL, NPS, agitation and symptoms of depression. Blinding to these measurements is not possible because these measurements require familiarity with the person’s usual behavior in the previous week(s). The nursing staff who complete the questionnaires are not blinded to intervention assignment, but they are not informed about the hypothesis and specific research questions.

#### Data analysis

Immediate effects are defined as the difference in change between outcomes 15 min before and after the intervention sessions. Cumulative effects are effects over time not including those immediately after the session (i.e., built-up effect over time and measured before the session in order not to include possible immediate effects of the session). Overall effects are effects over time including those immediately after the session. Immediate, cumulative and overall effects are analyzed with mixed model analysis to consider the dependency of the repeated observations within the patient.

For the immediate effects, the outcome will be the post-test (T1b, T2b) and adjustment will be made for the pre-test (T1a, T2a). For the cumulative effect, the outcome will be the pre-test and an adjustment will be made for the baseline. For overall effects, the outcome will be post-test and adjustment will be made for the baseline.

For all treatment effects, effects at the different time-points will be analyzed by adding a time x treatment interaction to the mixed model analysis. Furthermore, both crude and adjusted (adjustments for gender, age, cognitive deterioration and for medication use, which will be added as a time-dependent covariate) will be performed. In the mixed-model analysis, we will also evaluate whether an adjustment for the correlated observations within facilities is necessary. There is no need to impute missing data because mixed models can handle missing assessments.

In explorative post-hoc analysis we will evaluate whether the intervention effects will be different for patients with a musical background by adding the interaction between the intervention variable and presence of musical background.

Sleep quality data of the MotionWatch (CamNtech Ltd, Cambridge, UK) will be analyzed with Motionware software version 1.2.1. Five parameters will be calculated: Interdaily stability, Intradaily variability, Relative Amplitude, most active period of 10 h and least active period of five hours. The 5 parameters will be analyzed with linear regression analyses with the values measured after the intervention period as outcome with an adjustment for the baseline vale of particular outcome.

#### Study monitoring

This clinical trial does not involve a high-risk intervention. We do not expect adverse events or significant unintended effects of the intervention. However, the participants of this study are vulnerable subjects and monitoring, and quality assurance will be necessary according to national legislation (WMO). The Department of anesthesiology (an independent department of UMCG) will be responsible for the data monitoring.

## Discussion

Over recent decades, the care offered in nursing homes has expanded to include various types of psychosocial interventions such as music therapy, validation, gentle care, psychomotor therapy and movement activation [69]. Music therapy is a non-pharmacological intervention which is also used as a treatment for NPS in patients with dementia and can improve quality of life [70].

Quality of life (QoL) is a multi-dimensional concept that includes well-being and has objective and subjective effects [49–51]. Psychological well-being is considered to be the central indicator for QoL of patients with dementia [52]. This study aims to show if individual music therapy leads to improved well-being of nursing home residents with dementia and NPS. NPS result in a lower QoL for people with dementia and their caregivers and affect the quality of the relationship with the caregivers [71]. Efforts aiming at preventing low or decreasing well-being and QoL at any stage of dementia should, therefore, target the factors associated with lower QoL such as psychotropic drug use [17], NPS [9], pain [10] and sleep disturbances [15] as well as keeping a person-centered approach open to the individual perceptions of QoL [71]. Music-based interventions can be effective in alleviating the NPS of dementia, such as agitation, depression, and anxiety [72–74]. Recently, a systematic review concluded that non-pharmacological interventions such as music therapy show a positive impact on pain [75]. Further, a recent review of 8 studies identified 6 studies showing a positive effect of music interventions on sleep outcomes. There were decreases in nighttime sleep disturbance, increases in daytime alertness, and improvements in sleep quality [76].

Further, in patients unable to self-report, directly observed well-being is an expression of their emotional and physical state an important indicator of comfort and quality of life, in addition to impressions of quality of life assessed over a specific time window such as the previous week. Quality of life is an important outcome of different care approaches, including palliative care. Improving quality of life and maximizing comfort are goals of palliative care in dementia [77]. With this design and specific analyses, we can ensure blinding and allow estimation of short and long-term effects.

Participants receive either individual music therapy or a social visit for individual attention, in their own room. This is because the room represents a safe place for the resident. Moving residents to another room may lead unnecessary to more restless behavior during the already short duration of the therapy session.

Research often lacks methodological rigor and continues to produce mixed results. A recent Cochrane review [27] included 22 studies with 1097 randomized participants to assess the effects of music-based therapeutic interventions for people with dementia. This review showed modest positive effects in meta-analyses of 9 studies on emotional well-being and quality of life of interventions that included group and individual interventions. Only seven studies out of the 22 studies received individual music therapy treatment. Sensitivity analyses indicated that the SMDs for individual therapy were similar to those for the main analyses with individual and group therapy, except for behavioral problems (both agitation or aggression and overall), for which SMDs for individual therapy were clearly larger. In this Cochrane review, many studies did not used blinded outcome assessment. We use blinded assessment for the primary outcome (well-being) and one of the secondary outcome (pain) but possible limitations of this study will be the unblinded assessment of secondary outcomes (with the QUALID, NPI-NH, CMAI, CSDD) assessed by nurses of the department.

During the Covid-19 outbreak, the music therapists (intervention group) and the attendants (control group) used gloves and mouth masks through the sessions that may complicate the relation between participants and music therapists or attendants. Residents who participate in the study during Covid-19 period might receive substandard quality sessions because of the difficulties in the verbal and non-verbal contact. Sensitivity analysis will be done to examine whether this period with wearing of face masks and gloves yields a negative effect on treatment outcome, compared to the period before (or after) the outbreak of COVID-19.

The present study aims to make a next step in the field of music therapy and address the limitations of earlier research by including a larger number of participants and investigating if individual music therapy influences well-being of people with dementia and on NPS and other outcomes. Major strengths of the current study design are, first, the blinded primary outcome assessments and second, the comparison between intervention group with the control group receiving enhanced usual care to rule out effects of extra individual attention. The residents of the intervention group receive individual music therapy from qualified and certified music therapists with knowledge and skills that are clinically relevant to personalized care delivery. This study will help to clarify the effects of individual music therapy in dementia care.

## Data Availability

The handling of personal data is conducted according to the EU General Data Protection Regulation and the Dutch Act of Implementation of the General Data Protection Regulation (in Dutch: AVG, UAVG).

## Declarations

### Access to data

The first (VMBC) and fourth (SIMJ) authors will have access to the full dataset in Redcap. The anonymized data that support the findings of this study will be available upon reasonable request from the corresponding author. The data will not be publicity available due to privacy or ethical restrictions.

### Confidentiality

All study-related information will be stored securely at the study site. All participant information will be stored in locked file cabinets in areas with limited access. All files are confidential and coded with a 5-number code. The coding list is stored in a record cabinet with a key that only the first author can access.

## Abbreviations

NPS: Neuropsychiatric symptoms
MT: Music therapy
AMTA: American Music Therapy Association
NVvMT: Dutch association of music therapists:DS-DAT:Discomfort Scale - Dementia of Alzheimer Type
PAIC-15: Pain Assessment in Impaired Cognition
QUALID: Quality of Life in Late-Stage Dementia Scale
NPI-NH: Neuropsychiatric Inventory Nursing Home Version
CMAI: Cohen-Mansfield Agitation Inventory
CSDD: Cornell Scale for Depression in Dementia
GDS: Global Deterioration scale
WMO: Medical research involving human subjects act

## References

[1] World Health Organization Dementia. https://www.who.int/news-room/fact-sheets/detail/dementia (accessed 04 October 2021).

[2] Kester M, Scheltens P. Dementia. Practical neurology v9 n4 2009; 9:241–51. 10.1136/jnnp.2009.182477 19608778

[3] Zuidema SU, Verhey FRJ, Derksen E, Koopmans RTCM (2007). Prevalence of neuropsychiatric symptoms in a large sample of Dutch nursing home patients with dementia. Int J Geriatr Psychiatry.

[4] WHOQOL. Measuring Quality of Life. World Health Organization: https://www.who.int/publications/i/item/WHO-HIS-HSI-Rev.2012.03 (accessed 2 November 2022).

[5] Dodge R, Daly A, Huyton J, Sanders L (2012). The challenge of defining wellbeing. Int J Wellbeing.

[6] Clark C, Woods B, Moniz-Cook E, Mountain G, Øksnebjerg, Chattat R, Diaz A, Gove D, Vernooij-Dassen M, Wolverson E (2020). Measuring the well-being of people with dementia: a conceptual scoping review. BMC.

[7] Naglie G (2007). Quality of life in dementia. Can J Neurol Sci.

[8] Ettema TP, Droes R-M, de Lange J, Mellenbergh GJ, Ribbe. M. W: A review of quality-of-life instruments used in dementia. Qual Life Res. 2005; 14, *67*5–686. 10.1007/s11136-004-1258-0 16022061

[9] Wetzels RB, Zuidema SU, Jonghe JFM, Verhey FRJ, Koopmans RTC (2010). Determinants of quality of life in Nursing Home residents with dementia. Dement Geriatr Cogn Disord.

[10] Boerlage AA, van Dijk M, Stronks DL, de Wit R, van der Rijt CC (2008). Pain prevalence and characteristics in three Dutch residential homes. Eur J pain.

[11] Achterberg WP, Gambassi G, Finne-Soveri H, Liperoti R, Noro A, Frijters DH, Cherubini A, Dell’ Aquila G, Ribbe MW (2010). Pain in European long-term care facilities: cross-national study in Finland, Italy and the Netherlands. Pain.

[12] American Geriatrics Society Panel on Chronic Pain in Older Persons (1998). The management of chronic pain in older persons. J Am Geriatric Soc.

[13] Cipher DJ, Clifford PA (2004). Dementia, pain, depression, behavioural disturbances and ADLs: toward a comprehensive conceptualization of quality of life in long-term care. Int J Geriatr Psychiatry.

[14] Pieper MJ, van Dalen-Kok AH, Francke AL, van der Steen JT, Scherder EJ, Husebø BS, Achterberg WP. Interventions targeting pain or behaviour in dementia: a systematic review. Ageing Res Rev. 2013;12(4):1042–55. 10.1016/j.arr.2013.05.002. 10.1016/j.arr.2013.05.002 23727161

[15] Petrovsky DV, McPhillips MV, Li J, Caffee L, Hodgson NA (2018). Sleep disruption and quality of life in persons with dementia: a state-of-the-art review. Geriatr nurs (New York).

[16] van Iersel MB, Zuidema SU, Koopmans RT, Verhey FR, Olde Rikkert MG (2005). Antipsychotics for behavioural and psychological problems in elderly people with dementia: a systematic review of adverse events. Drugs Aging.

[17] Birkenhager-Gilesse EG, Kollen Bj, Achterberg WP, Boersma F, Jongman L, Zuidema SU (2018). Effects of Psychosocial interventions for behavioral and psychological symptoms in dementia on the prescription of psychotropic drugs: a systematic review and Meta analyses. J Am Med Dir Assoc.

[18] Olazaran J, Reisberg B, Clare L, Cruz I, Pena-Casanova J, Del ST, Woods B, Beck C, Auer S, Lai C, Spector A, Fazio S, Bond J, Kivipelto M, Brodaty H, Rojo JM, Collins H, Teri L, Mittelman M, Orrel M, Feldman HH, Muniz R (2010). Nonpharmacological therapies in Alzheimer’s disease: a systematic review of efficacy. Dement Geriatr Cogn Disord.

[19] Sikkes AMS, Tang Y, Jutten RJ, Wesselman LMP, Turkstra LS, Brodaty H, Clare L, Cassidy-Eagle E, Cox KL, Chételat G, Dautricourt S, Dhana K, Dodge H, Dröes RM, Hampstead BM, Holland T, Lampit A, Laver K, Lutz A, Lautenschlager NT, McCurry SM, Meiland FJM, Moris MC, Mueller KD, Peters R, Ridel G, Spector A, van der Steen JT, Tamplin J, Thompson Z, Bahar-Fuchs A. Toward a theory-based specification of non-pharmacological treatments in aging and dementia: focused reviews and methodological recommendations. J Alzheimer’s Association2021 02; 17(2): 255–70. 10.1002/alz.12188 PMC7970750 33215876

[20] Raglio A, Bellelli G, Mazzola P, Bellandi D, Giovagnoli AR, Farina E (2012). Music, music therapy and dementia: a review of literature and the recommendations of the Italian Psychogeriatric Association. Maturitas.

[21] Hsu MH, Flowerdew R, Parker M (2015). Jörg Fachner and Helen Odell-Miller. Individual music therapy for managing neuropsychiatric symptoms for people with dementia and their carers: a cluster randomised controleed feasibility study. BMC Geriatr.

[22] Ueda T, Suzukamo Y, Sato M, Izumi S (2013). Effects of music therapy on behavioral and psychological symptoms of dementia: a systematic reviwu and meta-analysis. Ageing Res Reviwua.

[23] American Music Therapy Association. Definition of Music Therapy. 2018. Available online: https://www.musictherapy.org/about/quotes/ Accessed on 11 September 2018.

[24] Hanser SB (2018). The New Music Therapist’s Handboek.

[25] Gold C, Eickholt J, Assmus J, Stige B, Wake JD, Baker FA, Tamplin J, Clark I, Lee YC, Jacobsen SL, Ridder HMO, Kretz G, Muthesius D, Wosch T, Ceccato E, Raglio A, Ruggeri M, Vink A, Zuidema S, Odell-Miller H, Orrell M, Scheneider J, Kubiak C, Romeo R, Geretsegger M. Music Interventions for Dementia and Depression in Elderly care (MIDDEL): protocol and statistical analysis plan for a multinational cluster-randomised trial. BMJ open 2019 03 30;9(3): e023436. 10.1136/bmjopen-2018-023436 PMC6475205 30928926

[26] Vink AC, Bruinsma MS, Scholten RJPM. Music therapy for people with dementia Cochrane database. Syst Rev. 2004;(3).CD003477. 10.1002/14651858.CD003477.pub2 15266489

[27] Van der Steen JT, Smaling HJA, van der Wouden JC, Bruinsma MS, Scholten R, Vink AC (2018). Music-based therapeutic interventions for people with dementia. Cochrane Database Syst Rev.

[28] Hanser SB (2016). Integrative Health through Music Therapy: accompanying the Journey from Illness to Wellness.

[29] Baroni Caramel V, Broersen M. Music therapy in dementia. Pilot about effect at neuropsychiatric symptoms Muziektherapie Bij dementia. Pilot naar het effect op neuropsychiatrische symptomen. [in Dutch] Tijdschrift voor Ouderengeneeskunde 2014,1:9–13.

[30] Sakamoto M, Ando H, Tsutou A (2013). Comparing the effects of different individualized music interventions for elderly individuals with severe dementia. Int Psychogeriatr.

[31] Saunders RP, Evans MH, Joshi P (2005). Developing a process-evaluation plan for assessing health promotion program implementation: a how-to guide. Health Promot Pract.

[32] Linnan L, Steckler A. Process Evaluation for Public Health Interventions and Research: an overview. Jossey-Bass: San Francisco; 2002.

[33] Cummings JL, Mega M, Gray K (1994). The neuropsychiatric inventory:comprehensive assessment of psychopathology in dementia. Neurology.

[34] Cummings JL (1997). The neuropsychiatric inventory: assessing psychopathology in dementia patients. Neurology.

[35] Wood S, Cummings JL, Hsu MA (2000). The use of the neuropsychiatric inventory in nursing home residents. Characterization and measurement. Am J Geriatr Psychiatry.

[36] Margallo M, Swann A, O’Brien J, Fairbairn A, Reichelt K, Potkins D, Mynt P, Ballard C (2001). Prevalence of the pharmacological and psychological symptoms amongst dementia suffers Iiving in care environments. Int J Geriatric Psychiatry.

[37] Zuidema SU, de Jonghe JFM, Verhey FRJ, Koopmans RTC (2010). Environmental correlates of neuropsychiatric symptoms in nursing home patients with dementia. Int J Geriatr Psychiatry.

[38] Leyhe T. July: Classification and overlap of depression and dementia. Alzheimer ‘s & Dementia v; 13 n7S_Part_28 (2017): P1346. 10.1016/j.jalz.2016.08.007 27693188

[39] Lyng K, Svingen E. Kartlegging av alvorlig, kombinerte sasetap hos eldre. Evaluering av en sjekklisterbasert screeingingmetodikk. Rapport 9/01. NOVA, Oslo, pp6.

[40] Roets-Merken LM, Zuidema ZU, Vernooij-Dassen MJFJ, Kempen GIJM. Screening for hearing, visual and dual sensory impairment in older adults using behavioural cues: a validation study. Int J Nurs Stud v51 nl 1 (2014): 1434–40. 10.1016/j.ijnurstu.2014.02.006 24656434

[41] Bruscia KE (1998). Defining music therapy.

[42] Guetin S, Charras K, Berard A, Arbus C, Berthelon P, Blanc F, Blayac JP, Bonte F, Bouceffa JP, Clement S, Ducourneau G, Gzill F, Laeng N, Lecourt E, Ledoux S, Plate) H, Thomas-Anterion C, Touchon J, Vrait FX, leger JM (2013). An overview of the use of music therapy in the context of Alzheimer’s disease: a report of a French expert group. Dementia.

[43] Gerdner LA, Schoenfelder DP (2010). Individualized music for elders with dementia. J Gerontol nurs.

[44] Raven-De Vries A, Verkerk M, Akse M, Denkbeeld. 2018, v30 n2 (201804):25–6.

[45] Kurstjens H. Observatieleidraad muzikale vormgeving, 2009.

[46] Lawton MP (1983). Environment and other determinants of well-being in older people. Gerontologist.

[47] Lawton MP, Lubben JE, Rowe JC, Deutchman DE (1991). A multidimensional view of quality of life in frail elders; in Birren JE. Quality of life in the frail Elderly.

[48] Lawton MP (1994). Quality of life in Alzheimer disease. Alzheimer Dis Assoc Disord.

[49] Jonker C, Gerritsen DL, Bosboom PR, van der Steen JT (2004). A model for quality-of-life measures in patients with dementia: Lawton’s next step. Dement Geriatr Cogn Disord.

[50] Hurley AC, Volicer BJ, Hanrahan PA (1992). Assessment of discomfort in advanced Alzheimer patients. Res Nurs Health.

[51] Hoogendoorn LI, Kamp Svd, Ader SMCA, Ooms HJ, van der Steen ME (2001). De Rol Van De Observator in De Betrouwbaarheid Van De Nederlandse Versie Van De Discomfort Scale- Dementia of Alzheimer Type (OS-DAT). Tijdschr Gerontol Geriatr.

[52] Van der Steen JT, Ooms ME, van der Wal G, Ribe MW (2002). Het meten van (on)welbevinden bij demente patienten. Tidschr Gerontol Geriatr.

[53] Van der Steen JT, Ooms ME, van der Wal G, Ribbe MW (2002). Het meten van (on)welbevinden bij dementia patienten - validiteit Van De Nederlandse Versie Van De Discomfort Scale- Dementia of Alzheimer Type (OS-DAT). Tijdschr Gerontol Geriatr.

[54] Corbett A, Achterberg W, Husebo B, Lobbezoo F, de Vet H, Kunz M, Strand L, Constantinou M, Tudose C, Kappesser J, de Waal M, Lautenbacher S. An international road map to improve pain assessment in people with impaired cognition: the development of the Pain Assessment in Impaired Cognition (PAIC) meta-tool. BMC Neurol; EU-COST action td I 005 Pain Assessment in Patients with Impaired Cognition, especially Dementia Collaborators: http://www.cost-tdl005.net/. 2014; 14:229. 10.1186/s12883-014-0229-5 PMC4279897 25726717

[55] Van Dalen-Kok AH, Achterberg WP, Rijkmans WE, Tukker-van Vuuren SA, Delwel S, de Vet HC, Lobbezoo F, de Waal MW (2017). Pain Assessment in impaired cognition (PAIC): content validity of the Dutch version of a new and universal tool to measure pain in dementia. Clin Interv Aging.

[56] Weiner MF, Martin-Cook K, Svetlik DA, Saine K, Foster B, Fontaone CS (2000). The quality of life in late-stage dementia (QUALID) scale. J Am Med Dir Assoc.

[57] Schalkwijk D, Verlare LR, Muller MT, Knol DL, van der Steen JT (2009). Het meten van kwaliteit van leven bij ernstig demente verpleeghuisbewoners: psychometrisch eigenschappen Van De Qualid-Schaal. Tijdschr Gerontol Geriatr.

[58] Moore DC, Payne S, Keegan T, Block L, Deliens L, Gambassi G, Heikkila R, Kijowska V, Pasman HR, Pivodic L, Froggatt K (2020). Length of stay in long-term care facilities: a comparison of residents in six European countries. Results of the PACE cross-sectional study. BMJ Open.

[59] Lange RT, Hopp GA, Kang N (2004). Psychometric properties and factor structure of the neuropsychiatric inventory nursing home version in an elderly neuropsychiatric population. Int J Geriatr Psychiatry.

[60] Kat MG, de Jonghe JFM, Aalten P (2002). Neuropsychiatric symptoms of dementia: psychometric aspects of the neuropsychiatric inventory (NPI) Dutch version. Tijdschr Gerontolol Geriatr.

[61] Cohen-Mansfield J, Billig N (1986). Agitated behaviour in the elderly 1. Conceptual review. J Am Geriatr Soc.

[62] Miller RJ, Snowdon J, Vaughan R (1995). The use of the Cohen-Mansfield Agitation Inventory in the assessment of behavioural disorders in nursing homes. J Am Geriatr Soc.

[63] De Jonghe JFM (1996). Factor structure and validity of the Dutch version of the Cohen Mansfield Agitation Inventory (CMAI-D). J Am Geriatr Soc.

[64] Alexopoulos GS, Abrams RC, Young RC (1988). Cornell Scale for Depression in Dementia. Biol Psychiatry.

[65] Muller-Thomsen T, Arlt S, Mann U, Mass R, Ganzer S. Detecting depression in Alzheimer’s disease: evaluation of four different scales. Arch Clin Neuropsychol. 2005;20(2):27 l-6. 10.1016/j.acn.2004.03.010 15708735

[66] Droes RM (1993). Cornell Scale for Depression in Dementia. Nederlandse vertaling. Interne publication.

[67] Reisberg B, Ferris SH, de Leon MJ, Crook T (1982). The global deterioration scale foe assessment of primary degenerative dementia. Am J Psychiatrie.

[68] Reisberg B, Ferris SH, Franssen E (1985). An ordinal functional assessment tool for Alzheimer’s-type dementia. Hosp Community Psychiatry.

[69] Dröes RM, Boelens-van der Knoop E, Bos J, Meihuizen L, Ettema T, Gerritsen D, Hoogeveen F, De Lange J, Schölzel-Dorenbos C. Quality of life in dementia in perspective. An explorative study of variations in opinions among people with dementia and their professional caregivers, and in literature. 2006, Dementia, 5, 4: pp. 533–58.

[70] Vasionnytė I, Madison G. May: Musical interventions for patients with dementia: a meta-analysis. Journal of clinical nursing v22 n9-10 (2013): 1203–16. 10.1111/jocn.12166 23574287

[71] Shin IS, Masterman D, Fairbanks L, Cummings JL (2005). Neuropsychiatric symptoms and quality of life in Alzheimer disease. Am J Geriatric Psychiatry.

[72] Ydstebø AE, Bergh S, Selbæk G, Šaltyte Benth J, Brønnick K, Vossius C, Haapala I, Biggs S, Kurrle S (2018). Longitudinal changes in quality of life among elderly people with and without dementia. Int Psychogeriatr.

[73] Guetin S, Portet F, Picot M, Pommié C, Messoudi M, Djabelkir L, Olsen AL, Cano MM, Lecourt E, Touchon J. Effect of music therapy on anxiety and depression in patients with Alzheimer’s type dementia: Randomized, controlled study. Dementia and geriatric cognitive disorders, v28 n1 (2009): 36–46. 10.1159/000229024 19628939

[74] Raglio A, Bellelli G, Traficante D, Gianotti M, Ubezio MC, Villani D, Trabucchi M. Efficacy of music therapy in treatment of behavioral and psychiatric symptoms of dementia. Alzheimer Disease and Associated disorders v22 n2 (2008):158–62. 10.1097/WAD.0b013e3181630b6f 18525288

[75] Liao YJ, Parajuli J, Jao YL, Kitko L, Berish D. Non-pharmacological interventions for pain in people with dementia: a systemic review. Int J Nurs Stud (2021), v124, 104082. 10.1016/j.ijnurstu.2021.104082 34607070

[76] Mu CX, Lee S, Boddupalli S, Meng H (2022). Effects of music interventions on sleep in people with dementia: a systematic review. Dementia.

[77] van der Steen JT, Radbruch L, Hertogh CMPM, de Boer ME, Hughes JC, Larkin P, Francke AL, Jünger S, Gove D, Firth G, Koopmans RTCM, Volicer L. White paper defining optimal palliative care in older people with dementia: a Delphi study and recommendations from the European Association for Palliative Care. Palliat Med. 201403;v28(n3):197–209. 10.1177/0269216313493685 23828874

